# Running a mobile diabetes screening service in Dominica

**Published:** 2020-12-31

**Authors:** Nanda Matthew

**Affiliations:** 1 Ophthalmic technologist and screener/grader for diabetic retinopathy: Ministry of Health, Roseau, Commonwealth of Dominica.

**An ophthalmic technologist and a nurse run an integrated diabetic retinopathy fundus photography screening service in the mountainous island of Dominica.**

**Figure:**
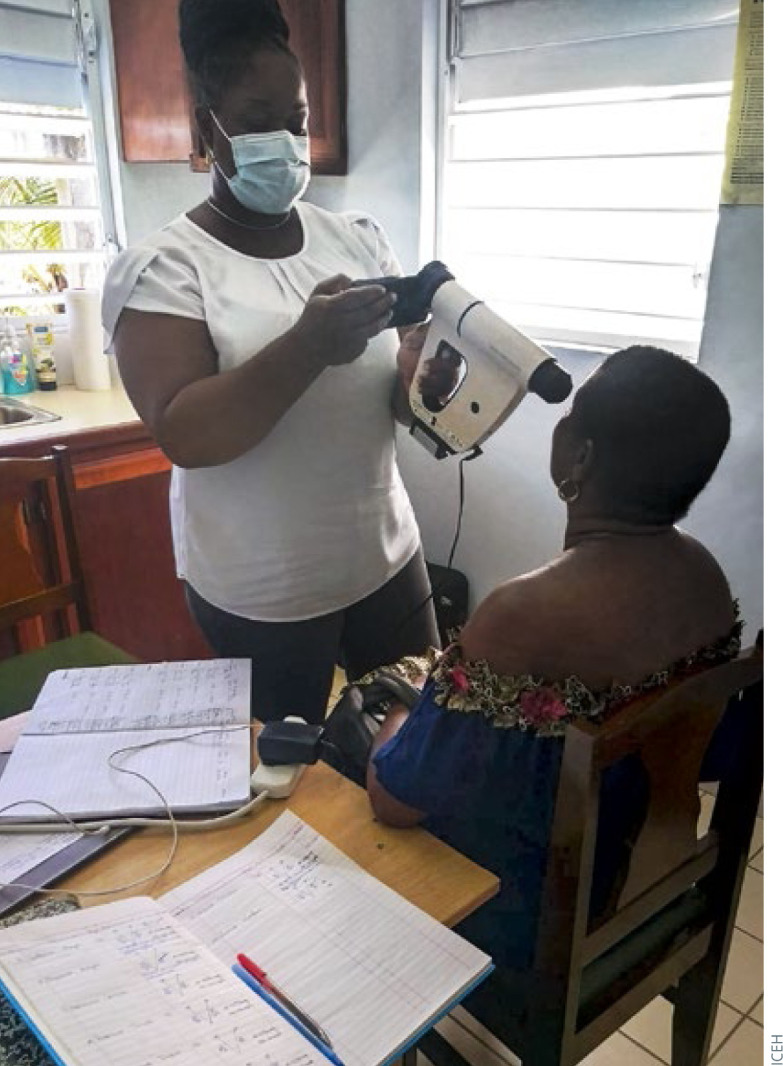
The author examines a patient during a mobile DR fundus photography screening clinic in the north of Dominica. **DOMINICA**

Dominica is a very mountainous island located in the Caribbean, with a population of about 72,000. Just over 10% of the population (7,500) are estimated to have diabetes mellitus and are at risk of developing diabetic retinopathy (DR), a condition that can lead to blindness if not detected and treated in time.

The geography makes it difficult for people with diabetes to travel to a central location for yearly fundus photography appointments that screen for DR. To address this need, a mobile DR screening clinic was added to the existing static screening clinic in the capital, Roseau, in 2005. The mobile clinic was expanded in 2016 thanks to funding from the Queen Elizabeth Diamond Jubilee Trust. The DR screening service in Dominica now has the capacity to screen around 6,500 people with diabetes per year and refer those showing signs of DR to an ophthalmologist as early as possible. Patients identified with other eye problems, especially cataract, large optic disc cups (where intraocular pressure (IOP) cannot be measured), or who need spectacles, are also referred to an ophthalmologist.

**“The geography makes it difficult for people with diabetes to travel to a central location for yearly diabetic retinophathy screening appointments.”**

Dominica (population 72,000) is divided into 7 primary health districts with 52 health centres, each of which is managed by a family nurse practitioner and senior community health nurses.

The country's DR screening service is run by two internationally certified screeners/graders: nurse assistant Carlene Luke Winston, responsible for the static clinic based at the China Friendship Hospital in Roseau, and Nanda Matthew, an ophthalmic technologist who runs the mobile clinic. Diabetes patients in Roseau Primary Health District (with 15 health centres serving around half of the population of Dominica) are screened at the static clinic. The mobile screening clinic serves the rest of the population by carrying out scheduled visits to 37 health centres in the remaining six districts; these are planned in collaboration with the team at each centre.

## The mobile screening clinic

The mobile screening clinics run from Monday to Thursday every week, and the screeners/graders prepare the schedule of visits to the health centres. The senior community health nurses at each centre are responsible for making appointments for the patients on their diabetes register, whether by word of mouth, telephone, church announcements or via the district medical officer's clinic. Patients are reminded to take their health books, sunglasses, a cap or an umbrella with them to their scheduled appointment in case there is a delay, and they are required to wait outside in the sun.

The health centres provide a well-lit room containing a table for the portable non-mydriatic fundus camera, a chair, and a Snellen visual acuity chart. Hand sanitiser must be available for use between patients.

At the start of each session, the screener/grader introduces herself to the patients and explains the screening procedure. The screener/grader then sees each patient individually to take a brief medical history, obtain consent, record functional distance visual acuity and instil dilating eye drops (phenylephrine 2.5%, tropicamide 1%). Patients return to the waiting area for about 20 minutes while their pupils dilate. When it's their turn to be screened, the screener/grader will warn them about the bright flash before capturing each image.

**Figure 1:**
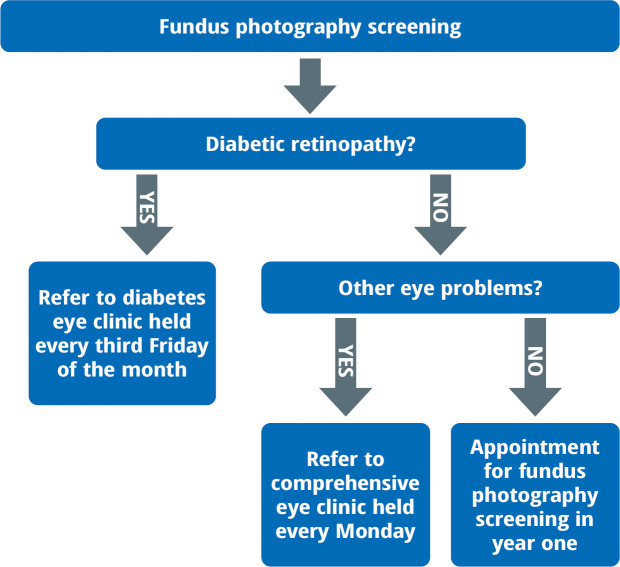
Referral protocols after diabetic retinopathy screening

Retinal fields are captured using a hand-held fundus camera, exported to the laptop, and graded immediately, as per protocol. The screener/grader then shows the image to each patient and explains the outcome.

## Referral

Patients with diabetic retinopathy are referred to a diabetes eye clinic. If a patient does not have diabetic retinopathy, or any other eye problems, an appointment is made for them to come back for screening in one year. Patients who do have other eye problems are referred to a comprehensive eye clinic, which is held once a week (Figure 1).

## Challenges

There are some challenges to overcome:

- Difficulty in communicating the clinic schedule to patients
- Scheduled clinics sometimes don't run due to staff absence, transportation issues and weather conditions (some health centres were damaged by Hurricane Maria and are no longer in use)
- Lack of a hydraulic table, which causes back strain when using the portable camera
- Lack of a tonometer to check patient's intraocular pressures
- A shortage of dilating eyedrops, which makes it difficult to capture good quality retinal images.

Public education needs to be ongoing to keep patients informed about the importance of screening. In addition, health care worker education is needed regularly to keep them motivated to refer people with diabetes for screening. This is particularly true for doctors in the private sector, who are not integrated into the country's health service. At least two more screener/graders and mobile cameras are needed to increase the number of new or follow-up patients seen in the clinics. Increasing capacity will, in turn, reduce the levels of blindness related to diabetic retinopathy and improve the quality of life for thousands of people living in Dominica.

